# A rare case of thymic carcinoid presenting with gastrointestinal symptoms and pericardium effusion

**DOI:** 10.1186/s12872-021-01871-4

**Published:** 2021-01-28

**Authors:** Qun-yan Xiang, Jin Xu, Ling Liu

**Affiliations:** 1 Department of Cardiovascular Medicine, The Second Xiangya Hospital, Central South University, Changsha, 410011 Hunan People’s Republic of China; 2 Research Institute of Blood Lipid and Atherosclerosis, Central South University, Changsha, 410011 Hunan People’s Republic of China; 3 Modern Cardiovascular Disease Clinical Technology Research Center of Hunan Province, Changsha, 410011 Hunan People’s Republic of China; 4 Cardiovascular Disease Research Center of Hunan Province, Changsha, 410011 Hunan People’s Republic of China

**Keywords:** Thymic carcinoid, Pericardium effusion, Ventricular invasion

## Abstract

**Background:**

Thymic carcinoid is one of an extremely rare type of malignant neuroendocrine tumor with a poor prognosis. Invasion of thymic carcinoid to other organs could lead to devastating consequences. It has been reported that thymic carcinoid mainly invaded to the pleura, lungs, liver, pancreas and bone, while rarely to the cardiac, especially to the ventricle.

**Case presentation:**

A 53-year-old man presented with gastrointestinal symptoms and persistent pericardial effusion. Multiple imaging tools, including chest computed tomography (CT), magnetic resonance imaging (MRI), 18F-Fluorodeoxyglucose positron emission tomography/CT (18F-FDG PET/CT) showed a malignant neoplasm arising from the thymus invading into the biventricular myocardium, pericardium, and left superior pulmonary veins. The tumor was finally diagnosed as a thymic carcinoid through pathological examination.

**Conclusion:**

This is a rare case of thymic carcinoid invading the ventricular myocardium, which presented as subacute heart failure. The observations in this case would be useful for differential diagnosis of primary heart disease and invasion of heart due to thymic carcinoid.

## Background

Thymic carcinoid is derived from the neuroendocrine system and was first described by Rosai et al. [1]. It is extremely rare with an incidence of 0.18 among 1,000,000 persons every year [2]. In addition to its rare occurrence, thymic carcinoid is often aggressive due to distant metastasis, invasive behavior and high rates of postoperative recurrence. Patients with thymic carcinoid are usually asymptomatic, which increases the difficulty of clinical diagnosis at an early stage.r A few of patients refer to hospital with endocrine disorders, including Cushing’s syndrome, multiple endocrine neoplasia types 1, and polyarthrosis due to the tumor secretes endocrine hormones, such as 5-hydroxytryptamine and prostaglandins [3, 4].

Invasion of thymic carcinoid to other organs is associated with potentially devastating consequences [5]. Metastasis to the pleura, lungs, liver, pancreas and bone has been reported in 20% of neoplasms [6], but cardiac invasion, mainly to the right atrium and pericardium has been rarely reported [7, 8]. Ventricular invasion of thymic carcinoid was extremely rare [9]. In this report, we describe a patient of thymic carcinoid with ventricular invasion, which offer a valuable insight for the diagnosis of thymic carcinoid.

## Case presentation

**Fig. 1:**
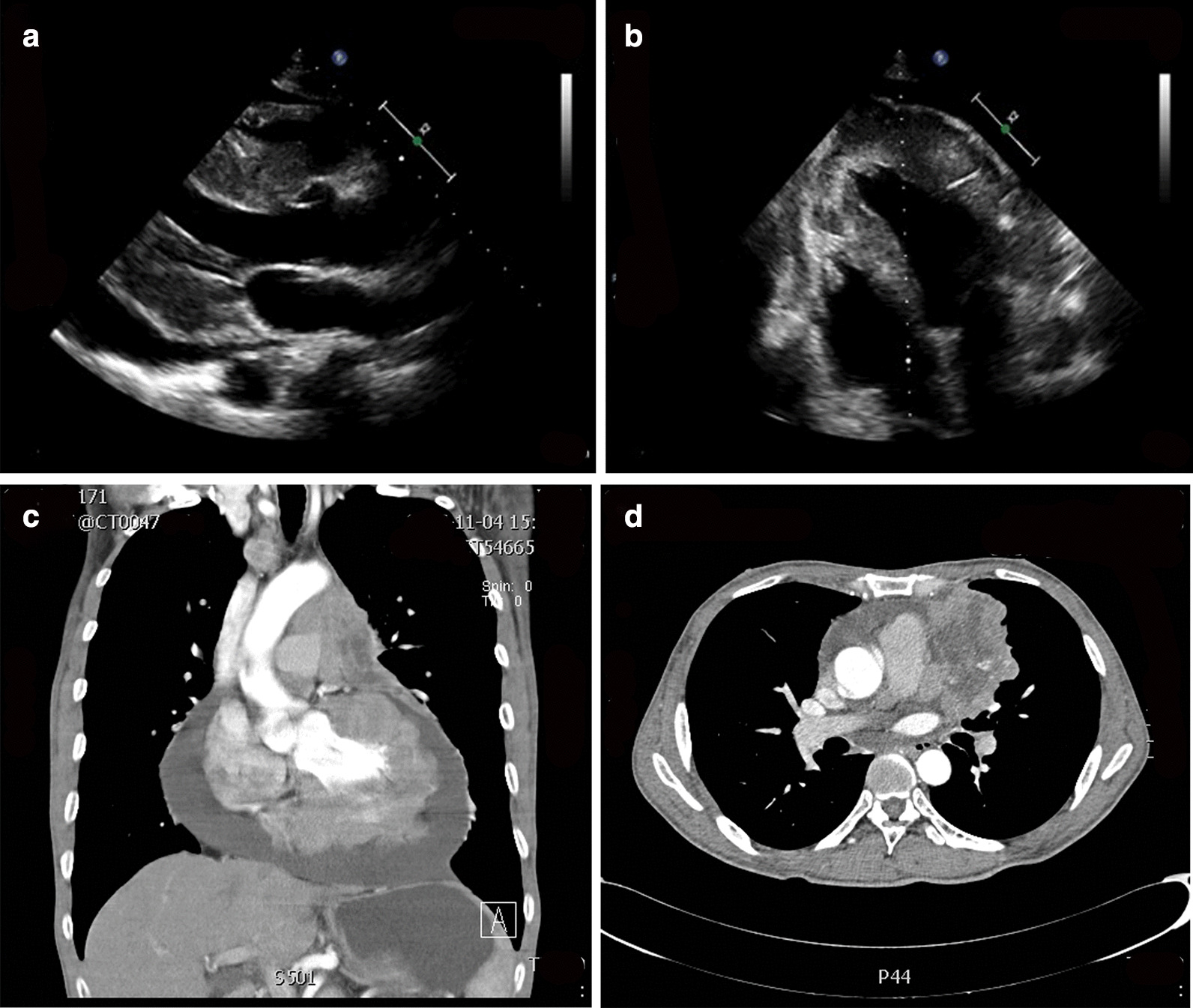
Echocardiography and chest CT scans. **a**, **b** Echocardiography showed severe pericardium effusion, thickened visceral pericardium, asymmetric septal hypertrophy, and thickened and nonhomogeneous ventricular walls with irregular echo-density of the myocardium. **c**, **d** CT scans showed an irregularly shaped mass (6.0 cm × 6.9 cm) in the anterior mediastinal region that had invaded the pericardium and left ventricular walls

A 53-year-old man presenting with persistent pericardial effusion, a 3-month history of cough, and weight loss of 7.5 kg within 2 years was referred to our hospital. Ten months ago, he had visited a local hospital for periumbilical pain, nausea, and diarrhea, where abdominal computed tomography (CT) scan showed the existence of pericardial effusion. Because of no symptoms of dyspnea or chest pain, the patients did not receive any treatment for pericardial effusion. He coughed in his supine position for 3 months, and then came to our hospital. In addition to severe pericardial effusion, symmetric septal hypertrophy (thickness 18–28 mm), thickened and nonhomogeneous left ventricular walls with irregular echo-density of the myocardium were also detected by echocardiography in our hospital (Fig. 1a, b). There was no sign of systolic anterior motion. Heart failure with preserved ejection fraction was considered for the following reasons: Firstly, the symptom of heart failure was supine dyspnea presenting as cough; Secondly, serum level of N-terminal pro-brain natriuretic peptide elevated to 19,005 pg/ml at the admission to our hospital; Thirdly, although the left ventricular ejection fraction was 62%, the left ventricular hypertrophy and diastolic dysfunction, presenting as decreased mitral annular velocity (6 cm/s) in the interventricular septum and free wall, were found. Thus, furosemide and spironolactone were prescribed to him.

**Fig. 2:**
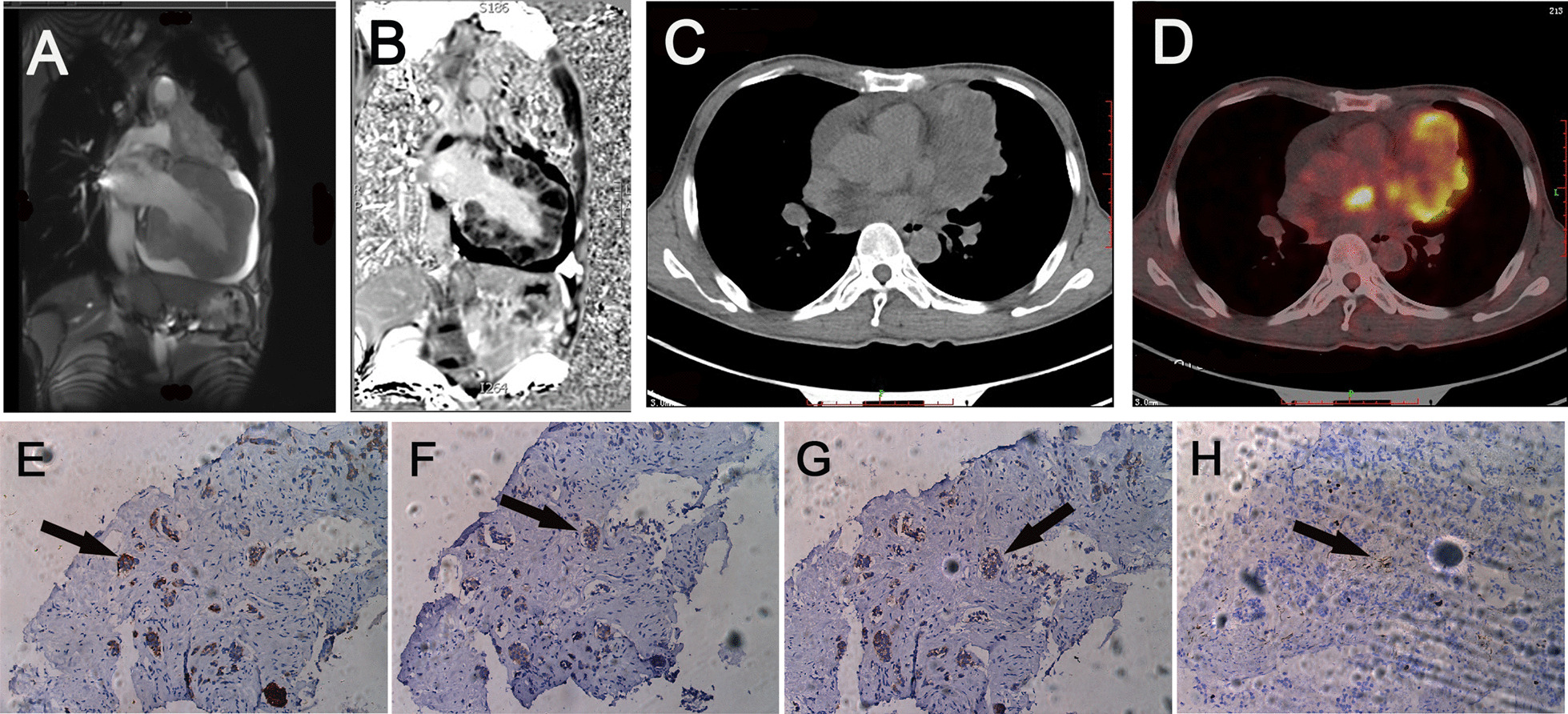
Cardiac MRI, total body 18F-FDG PET/CT scans and histopathologic of the tumor. **a**, **b** Cardiac MRI revealed hypointense signals on T1-weighted sequences, non-homogeneous hyperintense signals on T2-weighted sequences, and late gadolinium enhancement around the mass. **c**, **d** 18F-FDG PET/CT of the total body showed increased FDG uptake in the anterior mediastinal mass, multiple lymph nodes, ventricular walls, and left superior pulmonary veins. **e**–**h** Immunohistochemistry analysis showed that the tumor sample was positive for the neuroendocrine markers CgA (**e**), Syn (**f**), CD56 (**g**), and Ki-67 (5%) (**h**)

Chest CT showed an irregularly shaped and internal necrotic mass (6.0 × 6.9 cm), and multiple enlarged lymph nodes in the anterior mediastinal region. The mass had invaded the pericardium, left ventricular walls, and homolateral superior pulmonary veins (Fig. 1c, d). Magnetic resonance imaging (MRI) revealed hypointense signals on T1-weighted sequences, non-homogeneous hyperintense signals on T2-weighted sequences, and late gadolinium enhancement around the mass that was indicative of metastatic disease (Fig. 2a, b). Moreover, 18F-Fluorodeoxyglucose positron emission tomography/CT (18F-FDG PET/CT) of the total body revealed increased FDG uptake in the anterior mediastinal mass, multiple lymph nodes, left ventricular walls, and left superior pulmonary veins (Fig. 2c, d). Above findings strongly suggested an aggressive and malignant neoplasm arising from the thymus invading into the ventricular, pericardium, left superior pulmonary veins, and multiple lymph nodes.

Due to the superficial location of the thymic mass to the chest wall, CT-guided puncture was performed and specimen was taken for pathological biopsy. Hematoxylin eosin (HE) staining revealed the presence of a number of atypical, pleomorphic cells. Immunohistochemical staining showed the presence of the neuroendocrine markers synaptophysin (Syn), chromogranin A (CgA), the neuronal cell adhesion molecule CD56, and a proliferative index Ki-67 (5%) (Fig. 2e–h). Thus, the neuroendocrine origin of the thymic carcinoid was confirmed. The patient finally decided to give up further treatment due to personal choice.

## Discussion and conclusion

Thymic carcinoid is a rare type of neuroendocrine tumor, which accounts for 2–4% of all mediastinal tumors and only 0.4% of carcinoid tumors [10, 11]. Typically presenting at 4–5th decade, thymic carcinoid has a male dominance of about 3:1, with approximately a third of patients being asymptomatic [12]. A few of patients who become sick usually have chest pain, superior vena cava symptoms owing to their specific position in anterior mediastinal [3, 4, 13]. Due to their origin from neuroendocrine cells, about 50% of the tumors are functionally active through secretion of endocrine hormones, thus the patients often referred to hospital with endocrine disorders, including Cushing’s syndrome, multiple endocrine neoplasia types 1, and polyarthrosis [3, 14, 15]. More than 20% of the thymic carcinoid had signs of metastasis, mainly to pleura, lungs, liver, pancreas and bone [6]. Only a few cases with invasion to right atrium and pericardium were reported [7, 8], while there was extremely rare case with ventricular invasion [9]. In this report, we described a rare case of heart failure due to ventricular invasion of thymic carcinoid.

Roberto et al. [9] reported the neoplastic invasion of ventricular myocardium by a relapsing thymic carcinoid, showing several nodular capsulate lesions, thickened and nonhomogeneous ventricular walls and pericardium effusion, which were very similar to our case. But different from our case, the relapsed thymic carcinoid also invaded to other organs, such as lung, kidney and pancreas. There was no invasion of other organs except for the heart of this case in our hospital. Thus, it was difficult to distinguish metastasis from direct invasion. Because the thymus is adjacent to the heart, direct invasion is more likely than metastasis in this case. Anyhow, heart failure due to ventricular invasion of thymic carcinoid was extremely rare.

Diagnosis of thymic carcinoid on imaging examinations usually lack specificity. According to National Comprehensive Cancer Network guidelines, CT scanning is the initial choice for imaging modality [16]. Besides, PET/CT and MRI are also optional ways to determine the location of the tumor. CT and MRI typically demonstrate a mass of 2–20 cm in anterior mediastinal, with or without surrounding structures invasion [11]. The radiographs in this case showed an almost 7 cm mass in the anterior mediastinal region, which was consistent with the previous literature. The final diagnosis depends on pathology with immunohistochemically positive neuroendocrine markers include CgA, Syn and CD56 [17]. In our patient, histological tests and immunohistochemical staining showed all the above-mentioned neuroendocrine markers were positive, which confirmed the diagnosis of the tumor the originate of neuroendocrine system.

Radical surgery is the fundamental mode for the treatment of patients with thymic carcinoid. It has been reported that the prognosis was better in patients with surgery than those with conservative treatment, with a 5-year survival rate of 58% versus 26% [18]. According to National Comprehensive Cancer Network guidelines, chemotherapy with or without radiation therapy are recommended for unresectable or metastatic thymic carcinoid [16]. Considering the high risk of surgery and some personal reasons, the patient in this case decided not to perform any treatment such as operation, radiotherapy, or chemotherapy, but only follow-up.

In conclusion, our case highlights the rare occurrence of a thymic carcinoid invading the ventricular myocardium, which presented as subacute heart failure. The observations in this case would be useful for differential diagnosis of primary heart disease and invasion of ventricular due to thymic carcinoid.

## Data Availability

Data could be obtained upon request to the corresponding author.

## Abbreviations

CT: Chest computed tomography
MRI: Magnetic resonance imaging
PET/CT: Positron emission tomography/CT
18F-FDG PET/CT: 18F-Fluorodeoxyglucose positron emission tomography/CT
HE: Hematoxylin eosin
Syn: Synaptophysin
CgA: Chromogranin A

## References

[1] Rosai J, Higa E (1972). Mediastinal endocrine neoplasm, of probable thymic origin, related to carcinoid tumor. Clinicopathologic study of 8 cases. Cancer.

[2] Oberg K, Hellman P, Ferolla P (2012). Neuroendocrine bronchial and thymic tumors: ESMO Clinical Practice Guidelines for diagnosis, treatment and follow-up. Ann Oncol.

[3] Teh BT (1998). Thymic carcinoids in multiple endocrine neoplasia type 1. J Intern Med.

[4] Wick MR, Rosai J (1988). Neuroendocrine neoplasms of the thymus. Pathol Res Pract.

[5] Gaur P, Leary C, Yao JC (2010). Thymic neuroendocrine tumors: a SEER database analysis of 160 patients. Ann Surg.

[6] Mandegaran R, David S, Screaton N (2016). Cardiothoracic manifestations of neuroendocrine tumours. Br J Radiol.

[7] Ando Y, Hirabayashi N, Minami H (1995). Occult thymic carcinoma presenting as malignant cardiac tamponade. Intern Med.

[8] Promislow S, Dick A, Alzahrani A (2016). Recurrence of a thymic carcinoid tumour 15 years after resection with multiple myopericardial cardiac metastases: the role of multimodality imaging. Can J Cardiol.

[9] Bolognesi R, Vasini P, Tsialtas D (2001). Acute heart failure due to neoplastic invasion of ventricular myocardium by relapsing thymoma. Eur J Heart Fail.

[10] Ya Duh QY, Hybarger CP, Geist R (1987). Carcinoids associated with multiple endocrine neoplasia syndromes. Am J Surg.

[11] Yao JC, Hassan M, Phan A (2008). One hundred years after "carcinoid": epidemiology of and prognostic factors for neuroendocrine tumors in 35,825 cases in the United States. J Clin Oncol.

[12] Moran CA, Suster S (2000). Primary neuroendocrine carcinoma (thymic carcinoid) of the thymus with prominent oncocytic features: a clinicopathologic study of 22 cases. Mod Pathol.

[13] Sugawara K, Mizumoto M, Numajiri H (2014). Proton beam therapy for a patient with a giant thymic carcinoid tumor and severe superior vena cava syndrome. Rare Tumors.

[14] Dixon J, Borgaonkar S, Patel A (2013). Thymic neuroendocrine carcinoma producing ectopic adrenocorticotropic hormone and Cushing's syndrome. Ann Thorac Surg.

[15] Lowenthal RM, Gumpel JM (1974). Carcinoid tumour of the thymus with systemic manifestations: a radiological and pathological study. Thorax.

[16] Shah MH, Goldner WS, Halfdanarson TR (2018). NCCN guidelines insights: neuroendocrine and adrenal tumors, Version 2.2018. J Natl Compr Cancer Netw.

[17] Chaer R, Massad MG, Evans A (2002). Primary neuroendocrine tumors of the thymus. Ann Thorac Surg.

[18] Weksler B, Dhupar R, Parikh V (2013). Thymic carcinoma: a multivariate analysis of factors predictive of survival in 290 patients. Ann Thorac Surg.

